# Treating seronegative neuromyelitis optica spectrum disorder with inebilizumab: a case report

**DOI:** 10.3389/fneur.2023.1297341

**Published:** 2023-11-21

**Authors:** Dominik Lehrieder, Nikolaos Zapantis, Mirko Pham, Michael Klaus Schuhmann, Axel Haarmann

**Affiliations:** ^1^Department of Neurology, University Hospital Würzburg, Würzburg, Germany; ^2^Department of Neuroradiology, University Hospital Würzburg, Würzburg, Germany

**Keywords:** NMOSD, inebilizumab, AQP4, longitudinally extensive transverse myelitis, optic neuritis, case report, CD19, seronegative

## Abstract

**Background:**

Neuromyelitis optica spectrum disorder (NMOSD) is a devastating inflammatory disease of the central nervous system that is often severely disabling from the outset. The lack of pathognomonic aquaporin 4 (AQP4) antibodies in seronegative NMOSD not only hinders early diagnosis, but also limits therapeutic options, in contrast to AQP4 antibody-positive NMOSD, where the therapeutic landscape has recently evolved massively.

**Case presentation:**

We report a 56-year-old woman with bilateral optic neuritis and longitudinally extensive myelitis as the index events of a seronegative NMOSD, who was successfully treated with inebilizumab.

**Conclusion:**

Treatment with inebilizumab may be considered in aggressive seronegative NMOSD. Whether broader CD19-directed B cell depletion is more effective than treatment with rituximab remains elusive.

## Introduction

Neuromyelitis optica spectrum disorder (NMOSD), characterised by acute optic neuritis (ON) and longitudinally extensive transverse myelitis (LETM), is a rare autoimmune disease of the central nervous system. Compared with patients with multiple sclerosis, NMOSD patients tend to be older at onset, have more severe relapses, and remission is often incomplete, resulting in rapid progression of disability, highlighting the need for effective therapies. Relapse-independent accumulation of disability is rare. In most patients, a pathognomonic antibody against the water channel aquaporin 4 (AQP4) can be detected. Notably, myelin oligodendrocyte glycoprotein (MOG) antibodies can be detected in a subset of patients who are AQP4 antibody negative, suggesting a separate and well-defined disease entity that shares the clinical phenotype known as MOG antibody disease (1).

AQP4 is highly expressed on astrocytic end feet lining the abluminal cerebral vasculature. Binding of AQP4 antibodies (Ab) triggers an inflammatory response involving immune cell infiltration and activation of the classical complement pathway, culminating in the formation of a membrane attack complex and demyelinating lesions (2). Regions of high AQP4 expression (grey matter of the spinal cord, optic nerve) are clinically most affected. With the recent development of targeted monoclonal antibodies, including terminal complement inhibition (3), interleukin-6 pathway blockade (4, 5) and CD19-directed B cell depletion (6), the therapeutic landscape has certainly changed. While these drugs are approved for AQP4-positive NMOSD, none of the trials were designed to test efficacy in seronegative patients, who represent 20% of NMOSD patients.

Inebilizumab (INE) is an afucosylated IgG1 antibody directed against CD19. This modification of the Fc region results in optimised affinity for the Fc gamma receptor IIIA on leukocytes, significantly enhancing B-cell depletion due to increased antibody dependent cellular cytotoxicity (7). INE is given intravenously (induction with two infusions of 300 mg at day 1 and 15, maintenance therapy 300 mg every 6 months) and therefore has a high bioavailability. Given its size, INE is unlikely to penetrate the parenchyma as long as the endothelial barrier is intact. In contrast to CD20, CD19 is additionally expressed on pro-B cells, plasma blasts and plasma cells. By targeting a broader range of B cell subsets, INE is thought to cause more complete and rapid B cell depletion than traditional CD20 antibodies such as rituximab and ocrelizumab.

Interestingly, a post-hoc analysis of the double-blind, randomised, placebo-controlled phase 2/3 N-MOmentum trial suggests a treatment effect of INE in seronegative NMOSD. In this subgroup analysis by Marignier et al. 6 double seronegative patients showed a reduction in relapses at the individual level, although this was limited by the small sample size and did not withstand statistical testing (8).

To our knowledge, we are the first to report the successful treatment of a patient with seronegative NMOSD with the CD19 antibody INE in a real-world setting.

### Case description

**Figure 1:**
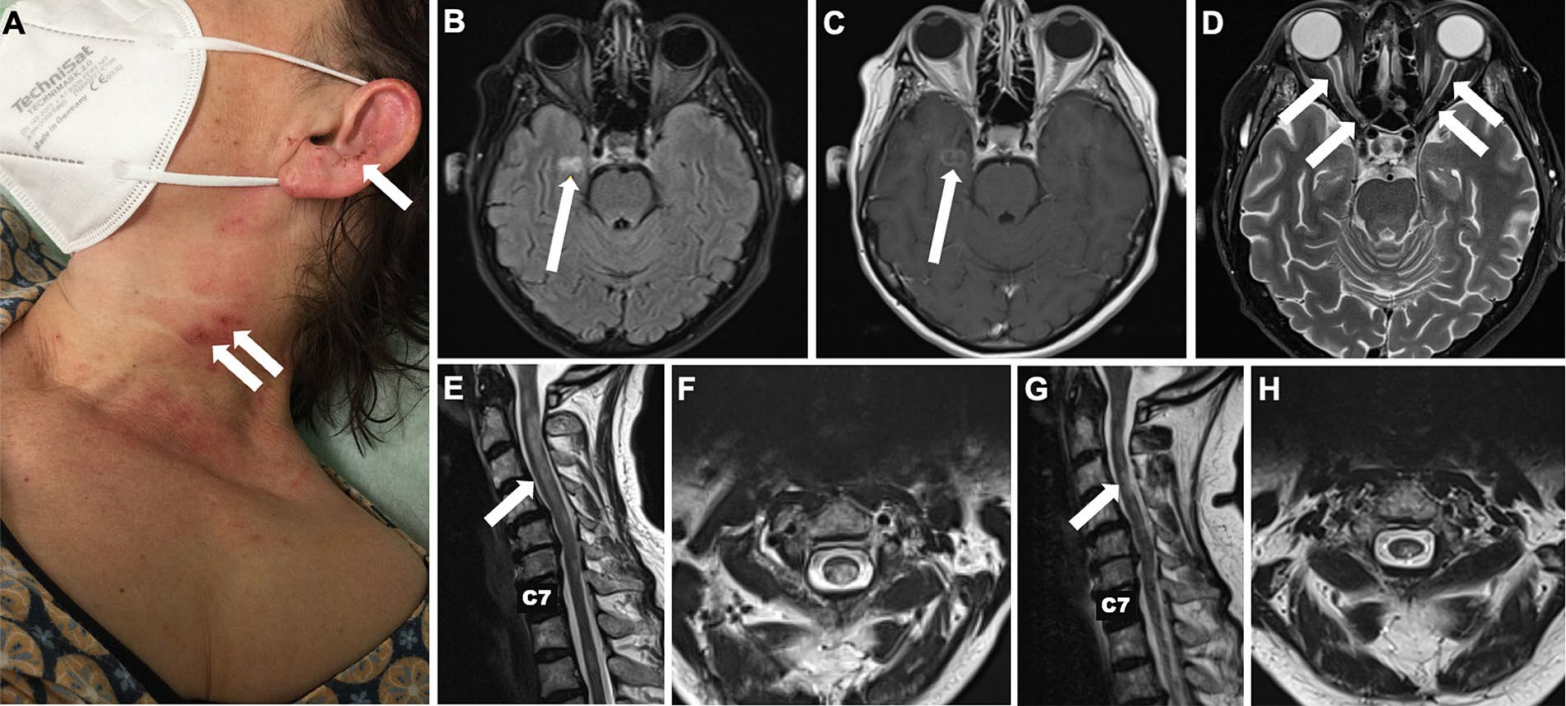
Skin changes and longitudinal MRI of brain and spinal cord. Localised painful rash with crusts on the neck and lateral face initially due to *repetitive scratching* in central neuropathic pain syndrome initially misdiagnosed as herpes zoster reactivation **(A)**. Cerebral T2-weighted FLAIR MRI showing a new hyperintense parahippocampal lesion that evolved within 6 days from symptom onset **(B)** and presented gadolinium enhancement **(C)**. T2 hyperintense lesions in both optic nerves consistent with optic neuritis **(D)**. Hyperintense spinal cord lesions compatible with cervical longitudinally extensive transverse myelitis reaching from the atlas to the 6th vertebra upon admission to our tertiary care centre **(E,F)** and after 6 months **(G,H)**.

A 56-year-old female patient was initially admitted to a secondary care centre. Her medical history included arterial hypertension, type 2 diabetes mellitus and breast cancer undergone full remission following treatment more than 15 years ago. The patient had just returned from a pilgrimage when she noticed a subacute sensorimotor deficit in her left hand. Initially physicians suspected a stroke, but the brain magnetic resonance imaging (MRI) showed no signs of acute or subacute ischemia or inflammation. She was admitted for further diagnostic evaluation. The next day she complained of blurred vision with no reported loss of visual acuity at that time. Over the next 3 days there was a marked clinical deterioration: The patient developed a vesiculopapular rash with secondary clustered crustation on her neck, ear and décolletage (Figure 1A) and became increasingly agitated and confused. Cerebrospinal fluid (CSF) examination revealed a mild pleocytosis of 10 [0–4] /μl. Empirical antiviral treatment with aciclovir was started on suspicion of herpes zoster encephalitis. However, subsequent polymerase chain reaction testing of the CSF was negative for varicella-zoster, herpes simplex and other neurotropic viruses. A follow-up cerebral MRI revealed a right parahippocampal T2 lesion that was not evident on the initial MRI (Figures 1B,C). In addition, there were T2 hyperintense lesions in both optic nerves, which were consistent with optic neuritis (Figure 1D). At this point the patient was referred to our tertiary care facility.

Here the patient was diagnosed with blindness in the right eye, severe reduction of visual acuity in the left eye (<0.1) and a marked ataxic tetraparesis. The rash, misdiagnosed as herpes zoster, turned out to be self-induced excoriation caused by the patient’s own fingers in an attempt to relieve a perceived severe burning and itching over the skin, which we attributed to a central pain syndrome, probably the cause of the agitation.

MRI of the spinal cord revealed spinal cord T2 hyperintense lesion locations and extension compatible with transverse myelitis (Figures 1E,F). A follow-up spinal tap was negative for oligoclonal bands. Chest/lung computed tomography scan revealed no evidence of pulmonary sarcoidosis. Initial testing for anti-AQP4 and anti-MOG antibodies was performed 12 days after clinical onset and prior to immunotherapy. Sera were analysed by cell-based indirect immunofluorescence at EUROIMMUN (Lübeck, Germany); staining at serum dilutions ≥1:10 was considered positive. Assays were negative at that time and at follow-up (Table 1). In contrast to AQP4-Ab-positive NMOSD, the diagnostic criteria for seronegative NMOSD are far more complex and require the presence of ≥2 core criteria, at least one of which must be ON, LETM or area postrema syndrome. Additional MR criteria must also be met. In our case, the patient had two core clinical features (bilateral ON and LETM) with brain MRI showing extensive (>1/2 optic nerve length) T2 hyperintense lesions of both optic nerves, a white matter lesion not suggestive of MS and acute myelitis involving more than 3 contiguous segments. In addition, differential diagnoses were ruled out as far as possible by repeated serological and CSF analysis. Thus, we diagnosed seronegative NMOSD according to the 2015 revised criteria of the International Panel for NMO Diagnosis (9). We started treatment with high-dose intravenous methylprednisolone at 1 g per day for 5 consecutive days. Due to lack of improvement, 7 sessions of plasma exchange were performed every other day with concomitant oral prednisolone therapy (60 mg/d) and prolonged tapering. The concomitant medications at that time were pantoprazole, L-thyroxine, liraglutide, basal insulin, naloxegol, pregabalin and mirtazapine.

**Table 1:** Dynamics of the laboratory findings at our tertiary care centre before and during INE therapy.

	Before high dose GC	Before induction of INE	3-month FU	6-month FU/second administration of INE	12-month FU/third administration of INE
Anti-AQP-4-Ab	Negative	Negative	Negative	–	–
Anti-MOG-Ab	Negative	–	Negative	–	–
IgG (mg/dl)	872	549	–	587	615
B cells (CD 19+)	–	22%	0.2%	0.0%	0.0%
T cells (CD3+)	–	72%	68%	75%	87%
Leukocytes *10^1000 γl	7.6	8.2	14.5	12.3	11.7
Neutrophils *10^1000 γl	5.06	5.26	12.39	10.06	9.01
Lymphocytes *10^1000 γl	1.80	2.27	0.70	1.02	1.30
Monocytes *10^1000 γl	0.61	0.47	0.71	0.97	1.07
CSF cells/mm^3^	13	3	–	–	–
CSF OCB	Negative	Negative	–	–	–

AQP-4-Ab, aquaporin-4 antibody; CSF, cerebrospinal fluid; FU, follow-up; GC, glucocorticoids; IgG, immunoglobulin G; INE, inebilizumab; MOG-Ab, myelin oligodentrocyte glycoprotein antibody; OCB, oligoclonal bands.

Given the recently approved therapies for AQP4-positive NMOSD and the fulminant onset of the disease, we decided to start INE as an off-label use. Administration of 300 mg on days 1 and 15 was well tolerated with no immediate or subacute adverse events. Prior to discharge to a rehabilitation clinic, the patient could recognise the basic shapes of most everyday objects with her left eye and was able to stand with much assistance.

**Figure 2:**
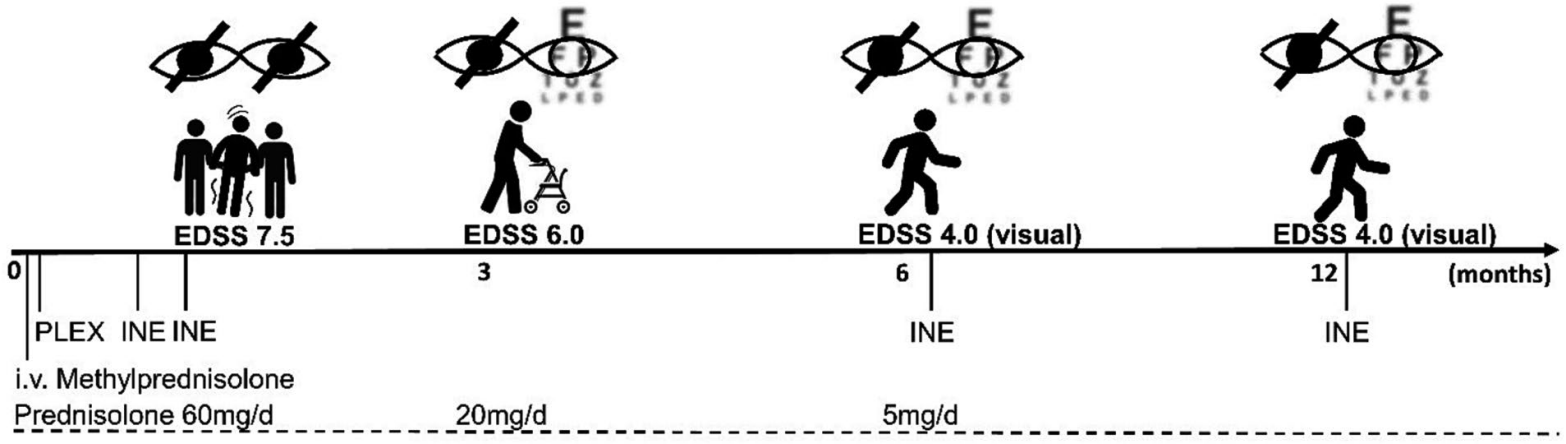
Timeline of the disease course. After initial high-dose i.v. methylprednisolone therapy, seven plasma exchange sessions (PLEX) were performed, and concomitant prednisolone was tapered to a dose of 5 mg/d over 6 months. The first cycle of INE infusions was administered a few days after PLEX, with a 14-day interval. The overall EDSS improved from 7.5 to 4.0 at 6 months and remained stable over the 12-month follow-up period, with persistent deficits mainly representing visual impairment.

At a follow-up visit 3 months later, the patient reported being able to walk up to 500 m with a walker. The right eye remained blind, colour vision had returned to the left eye, but she was still unable to read a newspaper. A brain MRI showed a new inflammatory T2 lesion in the left frontal white matter, possibly representing paraclinical disease activity before the immunotherapy could take full effect. Prednisolone was reduced from 20 mg to 5 mg/d. When we administered the second cycle of INE at the six-month follow-up, there were no new cerebral lesions and the inflammatory cervical myelopathy decreased in volume (Figures 1G,H). Walking was unrestricted; unfortunately, the visual impairment remained unchanged. Clinical stability was maintained after 12 months of treatment. A synopsis of the clinical course is shown in Figure 2.

## Discussion

First, our case illustrates that the potpourri of symptoms in NMOSD can be misleading, especially at the onset of the disease and for physicians unfamiliar with inflammatory disorders of the central nervous system. This is problematic because of the aggressive nature of the disease, where disabling clinical events require immediate and equally aggressive treatment (10). In this case, diagnosis was delayed by the lack of availability of an immediate MRI and neurological expertise in the secondary care clinic.

Besides diagnostic uncertainty due to lack of AQP4 antibodies, the case highlights the unmet need for tailored on-label therapies for patients with seronegative NMOSD.

Due to the severe first relapse, we hypothesised that this patient might benefit from the broader and rapid B cell depletion induced by INE: its effect can be seen within a week of administration. Although INE has only a half-life of about 18 days as it is eliminated by the reticuloendothelial system the effect of B cell depletion lasts for months (11, 12). By targeting CD19, INE also covers plasma blasts and plasma cells, as well as pro-B cells that do not express CD20 and would therefore be spared by rituximab. In fact, with regards to seropositive NMOSD, AQP4 antibodies have been shown to be secreted by a subpopulation of CD19-positive, CD20-negative plasma blasts that are increased during relapses in NMOSD patients (13).

In addition to antibody production, B cells can promote inflammation through cytokine secretion and as antigen-presenting cells, so broader depletion of autoreactive B cell populations by INE may also be more effective in seronegative NMOSD. Such superiority may be suggested by data from a further subgroup analysis of the N-MOmentum trial showing a reduction in relapse rate in patients with disease activity on rituximab after switching to INE. However, although including seronegative cases these patients were predominantly AQP4 Ab-positive (14). A comprehensive role for B cells in pathophysiology of NMOSD besides antibody production is supported by results of studies investigating interleukin-6 signalling pathways on NMOSD putting the relevance of plasma blasts in perspective: While Tocilizumab reduced relapse risk in seronegative and seropositive patients (15), Satralizumab was not beneficial in seronegative subgroup analysis (4). This suggests the relevance of additional pro-inflammatory pathways affecting disease activity as discussed above.

## Conclusion

To our knowledge, this is the first report of successful treatment with INE in a patient with double seronegative NMOSD outside of clinical trials. Although our experience with INE is encouraging, larger case series are clearly needed to show whether the presumed superiority of broader B cell depletion over earlier agents such as rituximab outweighs the additional cost. Randomised therapy trials focusing on seronegative NMOSD are tempting but are unlikely to yield reliable conclusions due to the inherent heterogeneity of the disease. Given the central role of B cells in the pathophysiology of NMOSD, it may be a reasonable option for physicians to consider (off-label) use of INE in patients with seronegative NMOSD, especially those with severe inflammation.

## Data availability statement

The datasets presented in this article are not readily available because of ethical and privacy restrictions. Requests to access the datasets should be directed to the corresponding author.

## Ethics statement

Written informed consent was obtained from the individual(s) for the publication of any potentially identifiable images or data included in this article.

## Author contributions

DL: Writing – original draft, Writing – review & editing. NZ: Writing – original draft, Writing – review & editing. MP: Writing – original draft, Writing – review & editing. MKS: Writing – original draft, Writing – review & editing. AH: Writing – original draft, Writing – review & editing.

## References

[1] Jarius S Ruprecht K Kleiter I Borisow N Asgari N Pitarokoili K. MOG-IgG in NMO and related disorders: a multicenter study of 50 patients. Part 2: epidemiology, clinical presentation, radiological and laboratory features, treatment responses, and long-term outcome. J Neuroinflammation. (2016) 13:280. doi: 10.1186/s12974-016-0718-0 27793206 PMC5086042

[2] Weinshenker BG Wingerchuk DM. Neuromyelitis Spectrum disorders. Mayo Clin Proc. (2017) 92:663–79. doi: 10.1016/j.mayocp.2016.12.014 28385199

[3] Pittock SJ Berthele A Fujihara K Kim HJ Levy M Palace J. Eculizumab in Aquaporin-4-positive Neuromyelitis Optica Spectrum disorder. N Engl J Med. (2019) 381:614–25. doi: 10.1056/NEJMoa1900866 31050279

[4] Traboulsee A Greenberg BM Bennett JL Szczechowski L Fox E Shkrobot S. Safety and efficacy of satralizumab monotherapy in neuromyelitis optica spectrum disorder: a randomised, double-blind, multicentre, placebo-controlled phase 3 trial. Lancet Neurol. (2020) 19:402–12. doi: 10.1016/S1474-4422(20)30078-8, PMID: 32333898 PMC7935419

[5] Yamamura T Kleiter I Fujihara K Palace J Greenberg B Zakrzewska-Pniewska B. Trial of Satralizumab in Neuromyelitis Optica Spectrum disorder. N Engl J Med. (2019) 381:2114–24. doi: 10.1056/NEJMoa1901747 31774956

[6] Cree BAC Bennett JL Kim HJ Weinshenker BG Pittock SJ Wingerchuk DM. Inebilizumab for the treatment of neuromyelitis optica spectrum disorder (N-MOmentum): a double-blind, randomised placebo-controlled phase 2/3 trial. Lancet. (2019) 394:1352–63. doi: 10.1016/S0140-6736(19)31817-3 31495497

[7] Ali F Sharma K Anjum V Ali A. Inebilizumab-cdon: USFDA approved for the treatment of NMOSD (Neuromyelitis Optica Spectrum disorder). Curr Drug Discov Technol. (2022) 19:e140122193419. doi: 10.2174/1570163818666210519103001, PMID: 34011260

[8] Marignier R Pittock SJ Paul F Kim HJ Bennett JL Weinshenker BG. AQP4-IgG-seronegative patient outcomes in the N-MOmentum trial of inebilizumab in neuromyelitis optica spectrum disorder. Mult Scler Relat Disord. (2022) 57:103356. doi: 10.1016/j.msard.2021.103356 35158465

[9] Wingerchuk DM Banwell B Bennett JL Cabre P Carroll W Chitnis T. International consensus diagnostic criteria for neuromyelitis optica spectrum disorders. Neurology. (2015) 85:177–89. doi: 10.1212/WNL.0000000000001729, PMID: 26092914 PMC4515040

[10] Jarius S Paul F Weinshenker BG Levy M Kim HJ Wildemann B. Neuromyelitis optica. Nat Rev Dis Primers. (2020) 6:85. doi: 10.1038/s41572-020-0214-9 33093467

[11] Bennett JL Aktas O Rees WA Smith MA Gunsior M Yan L. Association between B-cell depletion and attack risk in neuromyelitis optica spectrum disorder: an exploratory analysis from N-MOmentum, a double-blind, randomised, placebo-controlled, multicentre phase 2/3 trial. EBioMedicine. (2022) 86:104321. doi: 10.1016/j.ebiom.2022.104321, PMID: 36370634 PMC9664402

[12] Yan L Kimko H Wang B Cimbora D Katz E Rees WA. Population pharmacokinetic modeling of inebilizumab in subjects with neuromyelitis optica spectrum disorders, systemic sclerosis, or relapsing multiple sclerosis. Clin. Pharmacokinet. (2022) 61:387–400., PMID: 34718986 10.1007/s40262-021-01071-5 PMC8891208

[13] Chihara N Aranami T Sato W Miyazaki Y Miyake S Okamoto T. Interleukin 6 signaling promotes anti-aquaporin 4 autoantibody production from plasmablasts in neuromyelitis optica. Proc Natl Acad Sci U S A. (2011) 108:3701–6. doi: 10.1073/pnas.1017385108, PMID: 21321193 PMC3048150

[14] Flanagan EP Levy M Katz E Cimbora D Drappa J Mealy MA. Inebilizumab for treatment of neuromyelitis optica spectrum disorder in patients with prior rituximab use from the N-MOmentum study. Mult Scler Relat Disord. (2022) 57:103352. doi: 10.1016/j.msard.2021.103352, PMID: 35158461

[15] Ringelstein M Ayzenberg I Lindenblatt G Fischer K Gahlen A Novi G. Interleukin-6 receptor blockade in treatment-refractory MOG-IgG-associated disease and Neuromyelitis Optica Spectrum disorders. Neurol Neuroimmunol Neuroinflamm. (2022) 9:e1100. doi: 10.1212/NXI.0000000000001100, PMID: 34785575 PMC8596357

